# COVID-19-related psychological distress and engagement in preventative behaviors among individuals with severe mental illnesses

**DOI:** 10.1038/s41537-021-00136-5

**Published:** 2021-02-03

**Authors:** Amy E. Pinkham, Robert A. Ackerman, Colin A. Depp, Philip D. Harvey, Raeanne C. Moore

**Affiliations:** 1 School of Behavioral and Brain Sciences, The University of Texas at Dallas, Richardson, TX USA; 2 Department of Psychiatry, University of Texas Southwestern Medical School, Dallas, TX USA; 3 Department of Psychiatry, University of California San Diego, San Diego, CA USA; 4 VA San Diego Healthcare System, San Diego, CA USA; 5 Department of Psychiatry, University of Miami Miller School of Medicine, Miami, FL USA; 6 Research Service, Bruce W. Carter VA Medical Center, Miami, FL USA

**Keywords:** Schizophrenia, Psychosis

## Abstract

Individuals with severe mental illnesses (SMIs) may be disproportionately vulnerable to COVID-19 infection and psychological distress. This study investigated the prevalence of engagement in COVID-19 preventative behaviors, predictors of these behaviors, and COVID-19-related psychological distress. One hundred and sixty-three individuals with SMIs (94 with schizophrenia spectrum illnesses and 69 with affective disorders) and 27 psychiatrically healthy comparison participants were recruited from ongoing studies across 3 sites, to complete a phone survey querying implementation of 8 specific COVID-19 preventative behaviors that participants engaged in at least once in the past month as well as standard assessments of depression, anxiety, perceived stress, loneliness, and coping. Data were collected between 3 April 2020 and 4 June 2020. The large majority of our SMI sample, which consisted of outpatients with relatively mild symptom severity, endorsed engaging in multiple preventative behaviors. Relatively few differences were found between groups; however, individuals with SMI were less likely to work remotely than healthy individuals and individuals with schizophrenia spectrum illness were less likely to stay home as a preventative measure, wear face masks, and work remotely than individuals with affective disorders. Differences in staying home remained after controlling for potential confounds. Although individuals with SMI reported more psychological distress related to COVID-19, this distress was largely unrelated to engagement in preventative behaviors. The large majority of individuals with SMI in this outpatient sample, regardless of broad diagnostic category, reported performing multiple behaviors intended to prevent COVID-19 infection at least once a month and reported distress associated with the pandemic. These findings suggest a good level of awareness of COVID-19 among stable outpatients with SMI. The degree to which more acutely ill persons with SMI engage in such preventative behaviors, however, remains to be examined.

## Introduction

The novel coronavirus disease (COVID-19) has been reported in almost every country worldwide^1^ and has profoundly disrupted all aspects of society. Although effects of this pandemic are still unfolding, there is an acute need to assess and understand the impact of COVID-19 on marginalized populations who may be more vulnerable to poorer physical and mental health outcomes. Individuals with severe mental illnesses (SMIs) such as psychosis and affective disorders represent one such group^2,3^. For example, individuals with schizophrenia or bipolar disorder may be more susceptible to COVID-19 infection^4,5^ and, if infected, those with schizophrenia appear to be more than three times as likely than persons without schizophrenia to experience a severe clinical course^6^. Reasons for these disparities are many and varied but likely include greater vulnerability to stress^7,8^, reduced access to healthcare services^9,10^, increased prevalence of high risk factors such as obesity or diabetes^11,12^, and/or greater likelihood of communal living situations^13^ where rates of transmission could be increased.

In addition, individuals with SMI may be less aware of the pandemic and less likely to engage in preventative behaviors, which could lead to greater risk. A recent study conducted in India revealed that three quarters of individuals with SMI reported no fear or worry related to contracting COVID-19, and that only one quarter of patients were aware of three or more symptoms and/or precautionary strategies^14^. Greater educational achievement, socioeconomic status, and social support, as well as younger age, were all associated with greater COVID-19 knowledge^14^. Further, previous work investigating the attitudes of individuals with schizophrenia regarding the (H1N1)pdm09 flu virus found a reduced willingness to be vaccinated and to practice isolation among patients relative to non-mentally ill participants^15^. Individuals with schizophrenia were also less willing to wear a face mask, but this difference did not reach statistical significance^15^. These findings have prompted skepticism about the likelihood of individuals with SMI to engage in preventative behaviors in response to COVID-19^16^, but this has yet to be empirically investigated.

As described in Moore et al.^17^, our research team has three ongoing National Institute of Mental Health-funded studies that are specifically focused on SMI. These studies aim to understand how introspective accuracy contributes to functional outcomes in SMI (PI: Pinkham, R01 MH112620), how cognitive and social cognitive biases impact suicidal ideation and behavior in psychosis (PI: Depp, R01 MH116902), and how real-time mood affects in vivo cognitive performance and subsequent functioning in SMI (PI: Moore, R21 MH116104). These studies have cumulatively enrolled 247 individuals with diagnoses of schizophrenia, schizoaffective disorder, bipolar disorder (with or without psychotic features), or major depressive disorder with psychotic features, as well as a small sample (*n* = 31) of psychiatrically healthy comparison participants. In the current study, we contacted participants who were previously enrolled in these parent studies via phone and asked them to complete a verbal survey regarding utilization of preventative behaviors and the impact of COVID-19 on their current mental health. Survey responses were collected between 3 April 2020 and 4 June 2020 across three study sites, and a total of 163 individuals with SMI and 27 psychiatrically healthy comparison individuals responded. The key aims of this report are to identify the prevalence of engaging in specific preventative behaviors among individuals with SMI and to examine demographic and psychological factors (e.g., COVID-19-related anxiety), which may contribute to preventative actions. Based on recent data suggesting that individuals with affective disorders may be more affected by COVID-19-related stress relative to individuals with schizophrenia spectrum illnesses^18^, we also compared these broad diagnostic categories on preventative behaviors and current mental health.

## Results

### Responders vs. non-responders

To assess the possibility of self-selection bias, the group of survey responders (*n* = 190, comprised 163 SMI individuals and 27 healthy individuals) was first compared to those individuals from the parent studies, who did not respond to the survey (*n* = 88, comprised 84 SMI individuals and 4 healthy individuals). The responder group had a significantly higher percentage of healthy controls (14.2%) relative to the non-responder group (4.5%) (*χ*^2^(1) = 5.67, *p* = 0.017) and a higher percentage of females (59.4% responder vs. 43.2% non-responder; *χ*^2^(1) = 6.43, *p* = 0.011). There were no significant differences between responders and non-responders on race, ethnicity, employment status, relationship status, or residential status (all *p*s > 0.48). Responders and non-responders also did not differ in age (*t*(276) = 0.665, *p* = 0.51) or estimated IQ (*t*(246) = 0.32, *p* = 0.75); however, responders had completed more years of education (responder mean = 13.81, SD = 2.65 vs. non-responder mean = 12.62, SD = 2.49; *t*(276) = 3.55, *p* < 0.001).

For individuals with SMI in the responder and non-responder groups, there were no differences in specific diagnosis (*χ*^2^(4) = 6.57, *p* = 0.16) or broad diagnostic category (*χ*^2^(1) = 0.41, *p* = 0.52). Responders and non-responders with SMI also did not differ in the severity of any psychotic, mood, or insight symptoms assessed at baseline (all *ps* > 0.15).

### Preventative behaviors

The percentages of individuals from each group positively endorsing engaging in each preventative behavior are provided in Table 1. Individuals with SMI did not significantly differ from psychiatrically healthy controls on six of the eight behaviors, including self-distancing, avoiding public spaces/transportation, avoiding in-person visits with friends/family, staying home, handwashing/using sanitizer, and cleaning/disinfecting (all *φ* < 0.1; all *ps* > 0.21). However, a greater percentage of individuals in the SMI group reported wearing a face mask, and this difference was marginally statistically significant (*χ*^2^(1) = 3.68, *φ* = 0.14, *p* = 0.055). Further, healthy individuals were more likely to have worked or volunteered remotely (*χ*^2^(1) = 29.28, *φ* = 0.39, *p* < 0.001) and this difference remained significant when restricting the sample to only individuals who reported having some level of employment at their baseline visit (70.4% controls vs. 39.3% SMI; *χ*^*2*^(1) = 8.11, *φ* = 0.31, *p* = 0.004).

**Table 1:** Percentages of individuals positively endorsing engagement in COVID-19 preventative behaviors at least once in the past month.

Behavior	Psychiatrically healthy (*n* = 27)	Combined SMI group (*n* = 163)	Schizophrenia spectrum (*n* = 94)	Affective disorders (*n* = 69)
Self-distanced (chose to stay home and avoid contact with other people)	100	94.5	92.6	97.1
Worked or volunteered remotely	**70.4**	**20.2**	**10.6**	**33.3**
Avoided public spaces or public transportation	81.5	78.5	*73.4*	*85.5*
Avoided seeing friends and family members in person	66.7	71.8	72.3	71.0
Stayed at home, except for essential reasons (food, gas, medical)	81.5	84.0	**78.7**	**91.3**
Cleaned hands by handwashing or sanitizer	100	98.8	97.9	100
Used a face mask	*70.4*	*85.3*	**79.8**	**92.8**
Cleaned or disinfected frequently touched surfaces	81.5	82.8	83.0	82.6

Bold indicates a significant group difference at *p* < 0.05; italics indicate group differences that approached significance at *p* = 0.06.

Proportions of individuals with schizophrenia spectrum illnesses (i.e., schizophrenia and schizoaffective disorder) and those with affective disorders did not differ in positively endorsing self-distancing, avoiding in-person visits with friends/family, handwashing/using sanitizer, and cleaning/disinfecting (all *φ* < 0.1; all *ps* > 0.22). Individuals with affective disorders were more likely to use face masks (*χ*^*2*^(1) = 5.33, *φ* = 0.18, *p* = 0.02), to stay home as a precautionary measure (*χ*^*2*^(1) = 4.70, *φ* = 0.17, *p* = 0.03), and to work remotely (*χ*^*2*^(1) = 12.69, *φ* = 0.28, *p* < 0.001), which also remained significant when limiting the sample to those with reported employment (*χ*^*2*^(1) = 7.84, *φ* = 0.37, *p* = 0.005). There was also a trend for individuals with affective disorders to be more likely to avoid public spaces/transportation (*χ*^*2*^(1) = 3.46, *φ* = 0.15, *p* = 0.06).

### Psychological impacts

The SMI group reported greater depression, anxiety, perceived stress, loneliness, and worse coping than the psychiatrically healthy comparison group (all *ps* < 0.04; Table 2). However, there were no differences in these variables between individuals with schizophrenia spectrum illnesses and those with affective disorders (all *ps* > 0.23; Table 2).

**Table 2:** Group means on psychological impact assessments.

	Psychiatrically healthy (*n* = 27)		Combined SMI group (*n* = 163)		Cohen’s *d*
	Mean	SD	Mean	SD	
CES-D^a^	14.37	4.61	22.08	6.58	1.36
PROMIS-anxiety^a^	7.04	3.14	10.75	4.52	0.95
PSS^a^	8.93	2.70	10.77	3.68	0.57
UCLA-LS^a^	4.85	1.83	6.28	2.06	0.73
Brief COPE^a^	13.41	1.67	14.10	1.58	0.42

	Schizophrenia spectrum (*n* = 94)		Affective disorders (*n* = 69)		
CES-D	22.40	6.83	21.64	6.25	0.12
PROMIS-anxiety	11.11	4.95	10.28	3.85	0.19
PSS	10.69	3.83	10.88	3.50	0.05
UCLA-LS	6.35	2.06	6.13	2.06	0.11
Brief COPE	14.06	1.62	14.14	1.53	0.05

*Brief COPE* Brief Coping Orientation of Problems Experienced, *CES-D* Center for Epidemiologic Studies Depression Scale, *PROMIS-anxiety* Patient-Reported Outcome Measurement Information Systems Emotional Distress-Anxiety Scale, Short Form 4a, *PSS* Perceived Stress Scale, *UCLA-LS* University of California Los Angeles Loneliness Scale.

^a^Significant group difference at *p* < 0.05.

### Predictors of engaging in preventative behaviors

All demographic and clinical factors listed in Table 3 were examined (with the exception of diagnosis, which was addressed in the group comparisons above). Due to uneven distributions across categories, employment status was dichotomized as employed vs. unemployed, and race was reclassified into three categories: Caucasian, African American, and Other.

**Table 3:** Participant demographic and clinical characteristics.

Characteristic	Psychiatrically healthy (*n* = 27)		Combined SMI group (*n* = 163)		Schizophrenia spectrum (*n* = 94)		Affective disorders (*n* = 69)	
	*n*	(%)	*n*	(%)	*n*	(%)	*n*	(%)
Male^a^	13	48.1	64	39.3	44	46.8	20	29.0
Race^a^								
Caucasian	14	51.9	59	36.2	26	27.7	33	47.8
African American	4	14.8	65	39.9	52	55.3	13	18.8
Native American	0	0	2	1.2	1	1.1	1	1.4
Asian	4	14.8	8	4.9	2	2.1	6	8.7
Native Hawaiian/Pacific Islander	0	0	2	1.2	0	0	2	2.9
Other	5	18.5	27	16.6	13	13.8	14	20.3
Ethnicity^b^								
Hispanic	4	14.8	41	25.3	22	2.34	19	27.9
Non-Hispanic	23	85.2	121	74.7	72	76.6	49	72.1
Diagnosis								
Schizophrenia			41	25.2	41			
Schizoaffective			53	32.5	53			
Bipolar, psychotic features			38	23.3			38	
Bipolar, no psychotic features			30	18.4			30	
MDD, with psychotic features			1	0.6			1	
Employment Status^a,c^								
Employed, full time	18	66.7	19	11.7	3	3.2	16	23.2
Employed, part time	4	14.8	21	12.9	12	12.8	9	13.0
Unemployed	1	3.7	23	14.1	13	13.8	10	14.5
Stay-at-home parent	0	0	1	0.6	0	0	1	1.4
Part-time student	0	0	1	0.6	0	0	1	1.4
Full-time student	3	11.1	3	1.8	0	0	3	4.3
Receiving disability, unemp.	0	0	76	46.6	54	57.4	22	31.9
Receiving disability, part-time work	1	3.7	11	6.7	8	8.5	3	4.3
Retired	0	0	4	2.5	1	1.1	3	4.3
Relationship status^d,c^								
Single	15	55.6	94	57.6	57	62.6	37	54.4
In relationship	12	44.4	65	39.9	34	37.4	31	45.6
Residential status^d^								
Indep., financially responsible	25	92.6	108	67.9	57	62.6	51	75.0
Indep., not financially responsible	2	7.4	32	20.1	19	20.9	13	19.1
Residential facility, unsupervised	0	0	10	6.3	8	8.8	2	2.9
Residential facility, supervised	0	0	9	5.7	7	7.7	2	2.9

	Mean	SD	Mean	SD	Mean	SD	Mean	SD
Age (years)	38.41	12.24	42.74	11.26	43.18	10.79	42.14	11.93
Education (years)^a,^^c^	15.56	1.89	13.52	2.65	12.520	2.34	14.88	2.43
Estimated IQ^a,c^^,e^	105.83	5.97	97.40	11.27	94.31	10.28	102.24	11.12
PANSS positive total^a^^,e^			16.13	5.33	17.96	4.89	13.28	4.75
PANSS negative total^a^^,e^			12.08	3.69	13.12	3.98	10.45	2.43
PANSS general total^a^^,e^			30.46	7.53	31.45	8.09	28.91	6.30
MADRS^f^			12.67	11.10	12.00	11.21	13.56	10.97
YMRS^a^^,g^			2.36	4.68	1.43	3.72	3.56	5.48
SUMD^a^^,e^			4.07	1.56	4.44	1.75	3.48	0.96

*Indep.* independent, *Unemp.* unemployed.

^a^Schizophrenia Spectrum and Affective Disorders groups differ at *p* < 0.05.

^b^Ethnicity information was missing for 1 SMI individual.

^c^SMI and Healthy groups differ at *p* < 0.05.

^d^Information missing for 4 SMI individuals.

^e^WRAT, PANSS, and SUMD were not administered in one of the parent studies and information is therefore missing for 14 SMI individuals and 15 healthy individuals.

^f^Information missing for 5 SMI individuals.

^g^Information missing for 7 SMI individuals.

Engaging in self-distancing was significantly associated with increased educational achievement (*r* = 0.21; *p* = 0.008), as was working remotely (*r* = 0.34, *p* < 0.001). Endorsing working remotely was also associated with being employed (vs. unemployed) (*φ* = 0.34, *p* < 0.001), younger age (*r* = 0.26; *p* = 0.001), and higher IQ (*r* = 0.30; *p* < 0.001). The significant associations with education (*r* = 0.46; *p* < 0.001) and IQ (*r* = 0.46; *p* = 0.001) remained when restricting the sample to only individuals who were employed at baseline, and a novel relationship between ethnicity and working remotely emerged in this subsample. Ninety-five percent of individuals endorsing working remotely were non-Hispanic; in contrast, only 62% of those who were not working remotely were non-Hispanic (*φ* = 0.37, *p* = 0.006). Wearing a face mask was significantly associated with residential status such that higher proportions of those living independently (90% for independent and financially responsible, 84% for independent and not financially responsible) or in supervised settings (78%) reported wearing a face mask as compared to those residing in unsupervised residential facilities (50%) (Cramer’s *V* = 0.28, *p* = 0.006). Face mask use was also linked to lower negative symptom severity at baseline (*r* = 0.24; *p* = 0.003). Frequent cleaning/disinfecting of surfaces was only associated with race, with the highest percentage of endorsements among African Americans (95%), followed by Caucasians (78%), and then individuals in the Other category (69%) (Cramer’s *V* = 0.29, *p* = 0.001). Avoiding public spaces, avoiding in-person visits, staying home, and handwashing were not significantly associated with any demographic or clinical factors.

Of the eight specific preventative behaviors, only two were associated with psychological impact responses. Avoiding in-person visits was related to increased loneliness (*r* = 0.25; *p* = 0.002) and better coping (*r* = 0.22; *p* = 0.005), and staying home was associated with increased anxiety (*r* = 0.21; *p* = 0.008).

### Post-hoc analyses

To explore what may be contributing to diagnosis-based differences in preventative behaviors (i.e., working remotely, staying home, and using a face mask), we conducted logistic regression analyses to test whether the effect of diagnostic category remained significant after controlling for factors that were found to be related to that specific behavior. Differences between schizophrenia spectrum and affective disorders in working remotely were no longer significant after controlling for age, education, employment status, and estimated IQ (OR = 0.802 (95% CI: 0.277–2.32), *p* = 0.68) in the full sample, and after controlling for education, estimated IQ, and ethnicity in the subsample of those reporting employment at baseline (OR = 0.394 (95% CI: 0.079–1.96), *p* = 0.26). Group differences in using face masks also became nonsignificant after controlling for group differences in negative symptoms and residential status (OR = 0.440 (95% CI: 0.131–1.48), *p* = 0.18). However, differences in staying home remained significant after controlling for COVID-related anxiety, with individuals with schizophrenia spectrum illnesses being less likely than affective disorders to stay home (OR = 0.318 (95% CI: 0.118–0.858), *p* = 0.02).

Finally, given the potential for regional differences in the implementation of preventative behaviors, we examined site (The University of Texas at Dallas (UTD) vs. the University of Miami (UM) vs. University of California, San Diego (UCSD)) as a variable of interest in the analyses of the SMI group. Site differences were statistically significant only for working remotely (Cramer’s *V* = 0.25, *p* = 0.007) and wearing face masks (Cramer’s *V* = 0.29, *p* = 0.001). Thirty-three percent of the UCSD sample reported working remotely, whereas only 16% and 10% of the UTD and UM samples, respectively, reported the same. This site difference remained significant when limiting the sample to those who previously reported employment (percent working remotely at each site: UTD, 24%; UM, 13%; UCSD, 59%; Cramer’s *V* = 0.40, *p* = 0.011). In addition, despite the absence of mask mandates in all three states at the time of these surveys, face mask use was higher at UCSD (98%) than at UTD (75%) or UM (80%). As above, differences between schizophrenia spectrum and affective disorders in these behaviors remained nonsignificant when site was also included in the models (working remotely: OR = 0.879 (95% CI: 0.29–2.65), *p* = 0.81; face masks: OR = 0.545 (95% CI: 0.15–1.95), *p* = 0.35).

## Discussion

The current study investigated engagement in COVID-19 preventative behaviors, factors associated with performing preventative behaviors, and psychological responses to COVID-19 among individuals with SMI. Overall, a large majority of the SMI sample reported taking protective steps against COVID-19 infection, and with the exception of working remotely, the SMI sample endorsed engaging in preventative behaviors in approximately equal or greater numbers than the psychiatrically healthy control group. Given previous reports of potentially reduced awareness of COVID-19 among patients^14^ and reduced willingness to take precautionary measures against other pandemics^15^, these findings are particularly reassuring and suggest that clinically stable individuals with SMI may not represent a particularly vulnerable group. Despite finding differences in rates of working remotely, it is important to consider that many factors beyond the individual’s choice are likely to influence the ability to work remotely including the type/nature of the job and an employer’s willingness to allow remote work. Individuals in our sample may have chosen to work from home if possible and this group difference must therefore be interpreted cautiously.

Further, relatively few differences in preventative behavior engagement were found between SMI groups, but there were some exceptions. Individuals with affective disorders were more likely than individuals with schizophrenia spectrum illnesses to report working remotely, staying home, and wearing a face mask; although, only the group difference in staying home remained significant after controlling for demographic and clinical features that differed between groups. It is unclear why individuals with affective disorders were more likely to report staying home, in particular as only anxiety was significantly related to staying home and groups did not differ in reported anxiety; however, as with working remotely, this may be more related to opportunity than desire and additional work is needed to determine what influences a person’s options for staying home. Nevertheless, these findings may suggest somewhat reduced engagement in preventative behaviors among individuals with schizophrenia spectrum illness, which could translate to increased risk relative to individuals with affective disorders.

No factors emerged as consistent predictors of engaging in the various preventative behaviors. Higher educational attainment was the most prominent predictor, being associated with both self-distancing and working remotely; however, it was not associated with the other six preventative behaviors, suggesting somewhat limited importance overall. Surprisingly, COVID-related psychological distress showed only minimal relations to engaging in preventative behaviors. Increased anxiety was related to staying home, and increased loneliness and better coping were associated with avoiding in-person visits. However, the directionality of these relationships is unclear, given that reduced interaction with loved ones and the larger community likely contributes to anxiety and loneliness as well. We therefore cannot determine whether psychological distress is prompting preventative behaviors or vice versa. Of note, clinically assessed mood and psychotic symptoms at study baseline were also largely unrelated to preventative behaviors. Only increased negative symptoms were related to reduced use of face masks, which may suggest the potential for differential clusters of risk within the SMI group (e.g., prominent negative symptoms) that requires further investigation.

In terms of psychological responses to COVID-19, SMI individuals reported greater current psychological distress and reduced coping relative to heathy individuals. These differences are difficult to interpret given that SMI inherently involves increased psychological distress; however, the survey specifically queried the impact of COVID-19, suggesting that individuals with SMI may be disproportionately struggling with this pandemic. Moreover, contrary to data from an inpatient sample, suggesting more distress in affective disorders^18^, there were no differences in COVID-related depression, anxiety, stress, loneliness, or coping between diagnostic categories. It therefore may be helpful for clinicians to be broadly sensitive to COVID-related distress among all individuals with SMI.

Limitations of the current study include the small sample size for the psychologically healthy comparison group and the potential for self-selection bias. Although responders and non-responders did not differ on the majority of demographic factors or any clinical factors, responders were more likely to be female and to have more years of education relative to non-responders. Symptom severity for the full SMI sample was also relatively low, suggesting mild current illness. Our sample may therefore not be fully representative of those with SMI, and findings may not be generalizable beyond comparatively stable outpatients with SMI, and particularly not to those with acute illness. In addition, reduced variability and the relatively small number of individuals who reported not engaging in preventative behaviors may have contributed to the lack of systematic findings in regard to predictors of these behaviors. Relatedly, our strategy of querying preventative behaviors at any time in the last month did not allow us to assess the consistency with which individuals engaged in the behaviors, and the person-to-person format of the survey may have influenced individuals to respond in ways considered to be socially desirable. Both factors could have contributed to a potential ceiling effect in the preventative behavior data. Future work should attempt to recruit larger samples, examine the frequency and consistency with which individuals engage in these preventative behaviors, and utilize anonymous responding, all of which may reveal more variability.

The overall results indicate that in this sample of clinically stable outpatients with SMI, the majority of individuals, regardless of general type of SMI, are actively taking precautionary measures against COVID-19. These findings are in contrast to previous work on the (H1N1)pdm09 flu virus^15^ and speculations that reduced compliance with preventative behaviors may pose increased risk to communities and mental health clinicians^16^. Individuals with affective disorders appear to be slightly more proactive than individuals with schizophrenia spectrum illness, but both groups were largely comparable to healthy participants. Individuals with SMI are also reporting psychological distress associated with COVID-19; however, distress appears to be primarily unrelated to engaging in preventative behaviors. Although care should still be taken to ensure that the unique needs of individuals with SMI are met, the current results are encouraging and indicate that individuals with SMI are aware of the COVID-19 pandemic and are actively trying to stay safe.

## Methods

### Participants

Of the 278 individuals enrolled in our ongoing studies, 163 individuals with SMI and 27 psychiatrically healthy comparison individuals participated in the phone survey. Individuals in the SMI group were diagnosed with either schizophrenia, schizoaffective disorder, bipolar disorder (I or II) with or without psychotic features, or major depression with psychotic features. Following the convention of Kessler et al.^19^, the presence of psychotic features was deemed sufficient for classification as SMI, as was a diagnosis of bipolar I or II disorder. Diagnoses were determined at baseline study visits using the Mini International Neuropsychiatric Interview^20^ and the psychosis module of the Structured Clinical Interview for DSM-5^21^. Raters were trained on administration and scoring through videotape and practice interviews to acceptable inter-rater reliability (Intraclass Correlation Coefficient > 0.80). Demographic characteristics of the study sample are presented in Table 3.

SMI participants in the multi-site parent studies were recruited from the UCSD, UM, and UTD. Psychiatrically healthy comparison participants were recruited only from UTD (*n* = 12 responders, 4 non-responders) and UCSD (*n* = 15 responders). Recruitment procedures were similar across all sites, and both healthy and SMI participants were recruited through online advertisements and/or flyers at outpatient clinics. Inclusion and exclusion criteria varied slightly across studies, but in general, participants were adults aged 18 to 65 with estimated IQ > 70, as indicated by word reading performance on either the Wide Range Achievement Test-3 (WRAT-3)^22^ or the WRAT-4^23^. Healthy participants were free of psychiatric illness, and none of the SMI individuals were receiving inpatient care. All participants were free from neurological and/or neurodegenerative disorders. The institutional review boards at all three study sites approved the survey protocol and waived the requirement of written informed consent. Instead, all participants provided verbal consent for this survey, which included acknowledging that results would be published in aggregate. Full inclusion/exclusion criteria for each of the parent studies is provided in Supplementary Note 1.

### Measures

Baseline clinical characteristics, including mood and psychotic symptoms, were assessed at baseline visits of the parent studies, which occurred on average 225.89 days (SD = 124.81; range = 31–503) days prior to the phone surveys. The Positive and Negative Syndrome Scale^24^ was used to assess the severity of positive, negative, and general symptoms. Severity of depression symptoms was assessed using the Montgomery-Asberg Depression Rating Scale^25^ and severity of manic symptoms was assessed using the Young Mania Rating Scale^26^. The Scale to Assess Unawareness of Mental Disorder was used to evaluate insight into illness^27^. Descriptive statistics for the SMI group are provided in Table 1.

Engagement in preventative behaviors was assessed with eight yes/no questions targeting specific preventative/precautionary behaviors for COVID-19 that were developed for the current study. Participants were asked if they had engaged in each behavior at any time in the past month in an effort to prevent COVID-19 infection (Table 1).

The psychological impacts of COVID-19 were assessed via several open-source scales, including the Center for Epidemiologic Studies Depression Scale^28^, NIH PROMIS (Patient-Reported Outcome Measurement Information Systems) Emotional Distress-Anxiety Scale, Short Form 4a (PROMIS-anxiety)^29^, Perceived Stress Scale^30^, three items from the UCLA Loneliness Scale^31^, modified to be specific to experiences during the COVID-19 pandemic (e.g., “How often do you feel isolated from others during this period of COVID-19?”), and an 11-item brief coping scale (Brief COPE)^32^. For all assessments, higher scores indicated greater impact (i.e., more depression, worse coping, etc.).

### Statistical analyses

To assess representativeness of the current sample and the possibility of self-selection bias, demographic and baseline clinical characteristics were first compared between those individuals in the parent studies who completed the survey (i.e., responders) and those who did not (i.e., non-responders). Group differences in numbers of individuals endorsing each preventative behavior were then compared using *χ*^2^-tests. Comparisons were first made between patients and healthy controls and then between broad diagnostic categories: schizophrenia spectrum (schizophrenia and schizoaffective disorder) vs. affective disorders. Psychological impacts were also compared between both sets of groups using independent samples *t*-tests. Finally, to examine factors that may be related to engagement in each preventative behavior among individuals with SMI, associations with continuous variables were examined via point bi-serial correlations and correlations with categorical variables were examined via Phi or Cramer’s *V* as appropriate. To reduce the likelihood of type I error in these last analyses, only findings significant at *p* < 0.01 are reported.

### Reporting summary

Further information on research design is available in the Nature Research Reporting Summary linked to this article.

## Supplementary information

Supplemental Material

REPORTING SUMMARY

## Data Availability

The datasets generated and analyzed for the current study are available from the corresponding author upon reasonable request at amy.pinkham@utdallas.edu.

## References

[1] WHO. *WHO Coronavirus Disease (COVID-19) Situation Reports*. https://www.who.int/docs/default-source/coronaviruse/situation-reports/20200816-covid-19-sitrep-209.pdf?sfvrsn=5dde1ca2_2 (2020).

[2] Yao H, Chen J-H, Xu Y-F (2020). Patients with mental health disorders in the COVID-19 epidemic. Lancet Psychiatry.

[3] Maguire, P. A. & Looi, J. C. Vulnerability of people with schizophrenia to COVID-19. *Aus. N. Z. J. Psychiatry*. 10.1177/0004867420940775 (2020). 10.1177/0004867420940775 32615791

[4] Kozloff, N., Mulsant, B. H., Stergiopoulos, V. & Voineskos, A. N. The COVID-19 global pandemic: implications for people with schizophrenia and related disorders. *Schizophr. Bull.* **46**, 752–757 (2020). 10.1093/schbul/sbaa051 PMC7197583 32343342

[5] Stefana, A. et al. The COVID‐19 pandemic is a crisis and opportunity for bipolar disorder. *Bipolar Disorders* **22**, 641–643 (2020). 10.1111/bdi.12949 PMC7300615 32511859

[6] Ji, W. et al. Effect of underlying comorbidities on the infection and severity of COVID-19 in South Korea. *J. Korean Med. Sci.* **35**, e237 (2020). 10.3346/jkms.2020.35.e237 PMC7324262 32597048

[7] Norman RM, Malla AK (1993). Stressful life events and schizophrenia: I: a review of the research. Br. J. Psychiatry.

[8] Phillips LJ, Francey SM, Edwards J, McMurray N (2007). Stress and psychosis: towards the development of new models of investigation. Clin. Psychol. Rev..

[9] Bradford DW (2008). Access to medical care among persons with psychotic and major affective disorders. Psychiatr. Serv..

[10] Lawrence D, Kisely S (2010). Inequalities in healthcare provision for people with severe mental illness. J. Psychopharmacol..

[11] De Hert M (2011). Physical illness in patients with severe mental disorders. I. Prevalence, impact of medications and disparities in health care. World Psychiatry.

[12] Novak, P., Sanmartin, M. X., Ali, M. M. & Chen, J. Individuals with serious mental illness have more health conditions associated with severe illness from COVID-19. *Psychiatric Serv*. 10.1176/appi.ps.202000300 (2020). 10.1176/appi.ps.202000300 33208029

[13] Lee EE (2019). Comparison of schizophrenia outpatients in residential care facilities with those living with someone: study of mental and physical health, cognitive functioning, and biomarkers of aging. Psychiatry Res..

[14] Muruganandam, P., Neelamegam, S., Menon, V., Alexander, J. & Chaturvedi, S. K. COVID-19 and Severe Mental Illness: Impact on patients and its relation with their awareness about COVID-19. *Psychiatry Res*. **291**, 113265 (2020). 10.1016/j.psychres.2020.113265 PMC7322460 32763536

[15] Maguire PA, Reay RE, Looi JC (2019). Nothing to sneeze at–uptake of protective measures against an influenza pandemic by people with schizophrenia: willingness and perceived barriers. Australas. Psychiatry.

[16] Brown, E. et al. The potential impact of COVID-19 on psychosis: a rapid review of contemporary epidemic and pandemic research. *Schizophr. Res.* **22**, 79–87 (2020). 10.1016/j.schres.2020.05.005 PMC7200363 32389615

[17] Moore RC, Depp CA, Harvey PD, Pinkham AE (2020). Assessing the real-time mental health challenges of COVID-19 in individuals with serious mental illnesses: protocol for a quantitative study. JMIR Res. Protoc..

[18] Hölzle, P., Aly, L., Frank, W., Förstl, H. & Frank, A. COVID-19 distresses the depressed while schizophrenic patients are unimpressed: a study on psychiatric inpatients. *Psychiatry Res*. **291**, 113175 (2020). 10.1016/j.psychres.2020.113175 PMC7274101 32535514

[19] Kessler RC, Chiu WT, Demler O, Walters EE (2005). Prevalence, severity, and comorbidity of 12-month DSM-IV disorders in the National Comorbidity Survey Replication. Arch. Gen. Psychiatry.

[20] Sheehan DV (1998). The Mini-International Neuropsychiatric Interview (M.I.N.I.): the development and validation of a structured diagnostic psychiatric interview for DSM-IV and ICD-10. J. Clin. Psychiatry.

[21] First, M. B., Williams, J. B. W., Karg, R. S. & Spitzer, R. L. *Structured Clinical Interview for DSM-5—Research Version (SCID-5 for DSM-5, Research Version; SCID-5-RV)* (American Psychiatric Association, 2015).

[22] Wilkinson, G. S. *WRAT-3: Wide Range Achievement Test Administration Manual* (Wide Range, 1993).

[23] Wilkinson, G. S. & Robertson, G. J. *Wide Range Achievement Test 4 Professional Manual* (Psychological Assessment Resources, 2006).

[24] Kay SR, Fiszbein A, Opler LA (1987). The positive and negative syndrome scale (PANSS) for schizophrenia. Schizophrenia Bull..

[25] Montgomery SA, Åsberg M (1979). A new depression scale designed to be sensitive to change. Br. J. Psychiatry.

[26] Young RC, Biggs JT, Ziegler VE, Meyer DA (1978). A rating scale for mania: reliability, validity and sensitivity. Br. J. Psychiatry.

[27] Amador XF (1993). Assessment of insight in psychosis. Am. J. Psychiatry.

[28] Andresen E, Malmgren J, Carter W, DL P (1994). Screening for depression in well older adults: Evaluation of a short form of the CES-D. Am. J. Preventive Med..

[29] Cook K (2016). PROMIS measures of pain, fatigue, negative affect, physical function, and social function demonstrated clinical validity across a range of chronic conditions. J. Clin. Epidemiol..

[30] Cohen S, Kamarck T, Mermelstein R (1983). A global measure of perceived stress. J. Health Soc. Behav..

[31] Russell D (1996). UCLA loneliness scale (version 3): reliability, validity, and factor structure. J. Personal. Assess..

[32] Carver C (1997). You want to measure coping but your protocol’ too long: consider the brief cope. Int. J. Behav. Med..

