# Supplementary material for: COVID-19-related psychological distress and engagement in preventative behaviors among individuals with severe mental illnesses
Source: NPJ Schizophr. 2021 Feb 3;7:7. doi: 10.1038/s41537-021-00136-5 (PMC7859192; doi:10.1038/s41537-021-00136-5)
Supplement: Supplementary file 1 — Supplemental Material [file 41537_2021_136_MOESM1_ESM.pdf]

Supplementary Note 1

Participant Inclusion/Exclusion Criteria:

Parent Study 1 – All participants were: 1) between the ages 18 and 60, 2) proficient in English, and 3) able to provide informed consent. Additionally, all participants were required to have a high contact informant (with at least monthly encounters) who could provide data. Clinical participants were: 1) diagnosed with Bipolar I or II Disorder (with clinical stage of ≥ 3; current or past psychotic symptoms were not required) or schizophrenia/schizoaffective disorder, and 2) receiving stable pharmacotherapy (no medication changes for a minimum of 6 weeks, and no dose changes >20% for a minimum of two weeks).

Potential participants were excluded from the study if they had any of the following conditions: 1) presence or history of medical or neurological disorders that may affect brain function (e.g., stroke, epilepsy), 2) presence or history of neurodegenerative disorder (e.g. dementia, Parkinson's Disease), 3) history of unconsciousness for a period of greater than 15 minutes, 4) presence of sensory limitation including visual (e.g., blindness, glaucoma, vision uncorrectable to 20/40) or hearing (e.g. hearing loss) impairments that interfere with assessment, 5) presence or history of pervasive developmental disorder (e.g., autism) or mental retardation (defined as IQ <70) by DSM-5 criteria, 6) not proficient in English, 7) presence of substance dependence not in remission for the past six months. Additionally, healthy control participants could not meet past or present criteria for any psychiatric disorder.

Parent Study 2 – All participants were: 1) between the ages 18 and 65, 2) currently receiving outpatient treatment for schizophrenia, schizoaffective disorder, or one of the major mood disorders (Bipolar Disorder I, Bipolar Disorder II, or Major Depressive Disorder) *with psychotic features* according to DSM-5, 3) proficient in English, and 4) able to provide informed consent. Additionally, all participants were required to have a high contact informant (with at least monthly encounters) who could facilitate retention and safety.

Participants were excluded if they: 1) had experienced head trauma with loss of consciousness of >15 minutes, 2) had ever been diagnosed with neurological (e.g., stroke, epilepsy) or neurodegenerative disorder (e.g., dementia, Parkinson's Disease), 3) had sensory impairments that precluded assessments (e.g., vision or hearing problems), 4) had a diagnosis of intellectual disability (IQ<70), 5) were unable to arrive for screening or assessment in a non-intoxicated state, or 6) met criteria for substance use disorder, excluding cannabis and tobacco, within the past 3 months.

Parent Study 3 - All participants were: 1) between the ages 18 and 65, 2) proficient in English, and 3) able to provide informed consent. Clinical participants were: 1) diagnosed with Bipolar I or II Disorder, and 2) receiving only outpatient care.

Potential participants were excluded from the study if they had any of the following conditions: 1) presence or history of neurological disorder (e.g., seizures, Parkinson’s, stroke), 2) head trauma with unconsciousness > 30 minutes, 3) presence of sensory limitation including visual (e.g., blindness, glaucoma, vision uncorrectable to 20/40) or hearing (e.g. hearing loss) impairments that interfere with assessment, 4) presence of a DSM-5 Substance Use Disorder diagnosis in the past 3 months (excluding Cannabis Use Disorder), 5) current mood symptoms in the severe range (YMRS > 20 or MADRS > 30), or 6) active suicidal ideation as assessed by a score of 3 or higher on the Columbia Suicide Severity Rating Scale. Additionally, healthy control participants could not meet past or present criteria for any psychiatric disorder.
