# Supplementary material for: COVID-19-related psychological distress and engagement in preventative behaviors among individuals with severe mental illnesses
Source: NPJ Schizophr. 2021 Feb 3;7:7. doi: 10.1038/s41537-021-00136-5 (PMC7859192; doi:10.1038/s41537-021-00136-5)
Supplement: Supplementary file 2 — REPORTING SUMMARY [file 41537_2021_136_MOESM2_ESM.pdf]

nature research

Corresponding author(s): Pinkham, Amy E.

Last updated by author(s): Oct 30, 2020

# Reporting Summary

Nature Research wishes to improve the reproducibility of the work that we publish. This form provides structure for consistency and transparency in reporting. For further information on Nature Research policies, see our Editorial Policies and the Editorial Policy Checklist.

## Statistics

For all statistical analyses, confirm that the following items are present in the figure legend, table legend, main text, or Methods section.

| n/a | Confirmed | |
|---|---|---|
| ☐ | ☒ | The exact sample size ($n$) for each experimental group/condition, given as a discrete number and unit of measurement |
| ☐ | ☒ | A statement on whether measurements were taken from distinct samples or whether the same sample was measured repeatedly |
| ☐ | ☒ | The statistical test(s) used AND whether they are one- or two-sided<br>*Only common tests should be described solely by name; describe more complex techniques in the Methods section.* |
| ☐ | ☒ | A description of all covariates tested |
| ☐ | ☒ | A description of any assumptions or corrections, such as tests of normality and adjustment for multiple comparisons |
| ☐ | ☒ | A full description of the statistical parameters including central tendency (e.g. means) or other basic estimates (e.g. regression coefficient) AND variation (e.g. standard deviation) or associated estimates of uncertainty (e.g. confidence intervals) |
| ☐ | ☒ | For null hypothesis testing, the test statistic (e.g. $F$, $t$, $r$) with confidence intervals, effect sizes, degrees of freedom and $P$ value noted<br>*Give P values as exact values whenever suitable.* |
| ☒ | ☐ | For Bayesian analysis, information on the choice of priors and Markov chain Monte Carlo settings |
| ☒ | ☐ | For hierarchical and complex designs, identification of the appropriate level for tests and full reporting of outcomes |
| ☐ | ☒ | Estimates of effect sizes (e.g. Cohen's $d$, Pearson's $r$), indicating how they were calculated |

*Our web collection on statistics for biologists contains articles on many of the points above.*

## Software and code

Policy information about availability of computer code

| | |
|---|---|
| Data collection | *Provide a description of all commercial, open source and custom code used to collect the data in this study, specifying the version used OR state that no software was used.* |
| Data analysis | *Provide a description of all commercial, open source and custom code used to analyse the data in this study, specifying the version used OR state that no software was used.* |

For manuscripts utilizing custom algorithms or software that are central to the research but not yet described in published literature, software must be made available to editors and reviewers. We strongly encourage code deposition in a community repository (e.g. GitHub). See the Nature Research guidelines for submitting code & software for further information.

## Data

Policy information about availability of data

All manuscripts must include a data availability statement. This statement should provide the following information, where applicable:

- Accession codes, unique identifiers, or web links for publicly available datasets
- A list of figures that have associated raw data
- A description of any restrictions on data availability

The datasets generated and analyzed for the current study are available from the corresponding author upon reasonable request at amy.pinkham@utdallas.edu.

# Field-specific reporting

Please select the one below that is the best fit for your research. If you are not sure, read the appropriate sections before making your selection.

☐ Life sciences ☒ Behavioural & social sciences ☐ Ecological, evolutionary & environmental sciences

For a reference copy of the document with all sections, see nature.com/documents/nr-reporting-summary-flat.pdf

# Behavioural & social sciences study design

All studies must disclose on these points even when the disclosure is negative.

| | |
|---|---|
| Study description | Data are quantitative, cross-sectional survey responses. |
| Research sample | 163 individuals with severe mental illness (SMI; 94 with schizophrenia spectrum illnesses and 69 with affective disorders) and 27 psychiatrically healthy comparison participants. Approximately half of the sample was male, and average age was 39 years. |
| Sampling strategy | Participants were recruited from three parent studies of SMI, and 68.3% of individuals in those parent studies participated here. |
| Data collection | Data were collected via verbal surveys. Interviewers were blind to the specific study hypotheses. |
| Timing | Data were collected between April 3, 2020 and June 4, 2020. |
| Data exclusions | No data were excluded. |
| Non-participation | 31.7% of participants in the parent studies did not participate here. The vast majority (approximately 95%) of these individuals did not participate because we were unable to contact them. The remaining 5% were active refusals for various reasons (e.g., too busy). |
| Randomization | This was not an experiment and participants were therefore not randomized to a condition. |

# Reporting for specific materials, systems and methods

We require information from authors about some types of materials, experimental systems and methods used in many studies. Here, indicate whether each material, system or method listed is relevant to your study. If you are not sure if a list item applies to your research, read the appropriate section before selecting a response.

## Materials & experimental systems

| n/a | Involved in the study | |
|---|---|---|
| ☒ | ☐ | Antibodies |
| ☒ | ☐ | Eukaryotic cell lines |
| ☒ | ☐ | Palaeontology and archaeology |
| ☒ | ☐ | Animals and other organisms |
| ☐ | ☒ | Human research participants |
| ☒ | ☐ | Clinical data |
| ☒ | ☐ | Dual use research of concern |

## Methods

| n/a | Involved in the study | |
|---|---|---|
| ☒ | ☐ | ChIP-seq |
| ☒ | ☐ | Flow cytometry |
| ☒ | ☐ | MRI-based neuroimaging |

## Human research participants

Policy information about studies involving human research participants

| | |
|---|---|
| Population characteristics | See above. |
| Recruitment | Participants were recruited through online advertisements and/or flyers at outpatient clinics. Survey responders were more likely to be female and to have more years of education relative to non-responders. Our sample may therefore not be fully representative of those with SMI and findings may not be generalizable beyond comparatively stable outpatients with SMI. |
| Ethics oversight | The IRBs of the University of California, San Diego (UCSD), the University of Miami (UM), and The University of Texas at Dallas (UTD). |

Note that full information on the approval of the study protocol must also be provided in the manuscript.
